# Designing a Person-Centred Integrated Care Programme for People with Complex Chronic Conditions: A Case Study from Catalonia

**DOI:** 10.5334/ijic.5653

**Published:** 2021-10-01

**Authors:** Miquel À. Mas, Ramón Miralles, Consol Heras, Maria J. Ulldemolins, Josep M. Bonet, Núria Prat, Mar Isnard, Sara Pablo, Sara Rodoreda, Joaquim Verdaguer, Magdalena Lladó, Eduard Moreno-Gabriel, Agustín Urrutia, Maria A. Rocabayera, Nemesio Moreno-Millan, Josep M. Modol, Isabel Andrés, Oriol Estrada, Jordi Ara Del Rey, ProPCC-Badalona Group, Salvador Altimir

**Affiliations:** 1 Direcció Clínica Territorial de Cronicitat Metropolitana Nord, Institut Català de la Salut, Catalonia, Spain; 2 Department of Geriatrics, Hospital Universitari Germans Trias i Pujol, Badalona, Catalonia, Spain; 3 Universitat Autònoma de Barcelona, Catalonia, Spain; 4 Direcció d’Atenció Primària Metropolitana Nord, Institut Català de la Salut, Catalonia, Spain; 5 Servei d’Atenció Primària Barcelonès Nord i Maresme, Institut Català de la Salut, Badalona, Catalonia, Spain; 6 Hospital Universitari Germans Trias i Pujol, Badalona, Catalonia, Spain; 7 Gerència Territorial Metropolitana Nord, Institut Català de la Salut, Catalonia, Spain; 8 All members affiliated to Institut Català de la Salut except Margarita Álvaro, affiliated to Institut Català d’Oncologia.

**Keywords:** integrated care, personcentredness;complex chronic conditions;programme design, multimorbidity, advanced conditions, older people

## Abstract

**Introduction:**

The prevalence of people with complex chronic conditions is increasing. This population’s high social and health needs require person-centred integrated approaches to care.

**Methods:**

To collect data about experiences with the health system and identify priorities for care, we conducted 2 focus groups and 15 semi-structured interviews involving patients with multimorbidity and advanced conditions, caregivers, and representatives of patients’ associations. To design the programme, we combined this information with evidence-based recommendations from local healthcare and social care professionals.

**Results:**

Patients’ and caregivers’ main priorities were to ensure (a) comprehension of information provided by healthcare professionals; (b) coordination between patients, caregivers, and professionals; (c) access to social services; (d) support to caregivers in managing situations; (e) perceived support throughout the healthcare process; (f) home care, when available; and (d) a patient-centred approach. These dimensions were included in 37 of 63 clinical actions of the programme to cover the whole care trajectory: identifying high needs, defining, and providing care plans, managing crises, and providing transitional care and end-of-life care.

**Conclusion:**

We developed an evidence-based integrated care programme tailored to high-need patients combining input from patients, caregivers, and healthcare and social care professionals.

## Introduction

Societal ageing and decreased mortality associated with various conditions due to improved health-system efficiency have led to an increase in the number of frail older people and people with multimorbidity [1]. Various studies show there is a small group of people with complex chronic conditions (such as multimorbidity, advanced frailty or advanced illness) who are characterized by high health and social needs. This population, often referred to as “high-need, high-cost patients” [2], requires a person-centred approach; otherwise, their needs can go unmet when fragmented care fails to cover one or more conditions [3,4]. This multiple needs coverage require an integrated approach; thus, it is imperative to develop and improve integrated care models to ensure the health and social needs are met [5,6,7,8,9,10].

In designing these models to move from disease-centred approaches to person-centred integrated care, it is crucial to take people experience into consideration [11,12,13,14,15]. Various initiatives in recent years have recommended exploring people priorities and asking them what matters most [16,17]. Furthermore, researchers have identified system-related and professional-related issues that should be considered in planning healthcare services for these people [18] and have identified key aspects related to communication/information, care management, and care coordination that should be included [19].

### Background of this Research

Our purpose was contextualised in the framework of the Health Plan of Catalonia [20]. In this context, the Catalan Chronic Care Programme was led in 2011 with a focus on improving the care of patients with chronic conditions. Main actions included: (a) to stratify the entire Catalan population and to identify risk groups to work proactively with them; (b). to establish a model for complex chronic and advanced chronic conditions identification that generates shared intervention plans; (c). to define and implement a proactive care and case management model; (d). to introduce a palliative care approach to people with advanced chronic conditions; (e). to design and implement a collaborative health and social model for people with chronic conditions and higher levels of dependence; (f). to implement a new complex chronic care model for assessment and contracting; (g). to guarantee health and social integrated care for people with both complex chronic conditions and social care needs. Last decade, different local providers adapted their strategies to identify high need people to tailor care plans to two targeting populations derived from this programme [21]: complex chronic patients (CCP) and patients who had advanced chronic disease (ACD). Several clinical programmes have been implemented across the territory to develop and assess intervention strategies, following this identification [22].

### Aims and Objectives of the Engagement Process

Following the strategy of the Government of Catalonia, Institut Català de la Salut, as main health public provider in our region, have developed a proactive strategy to identify needs of people with complex chronic conditions. To consolidate a standardised care model for these populations, there were found as key actions not only to target people with high health and social needs, but also to involve all actors in shared care plans. Thus, in designing a person-centred integrated care programme, reference for the care of people with complex chronic conditions in our institution, we aimed to draw on people’s experience with healthcare to identify key clinical actions in order that their priorities and views could be included in the programme, integrating them with evidence-based clinical actions proposed by clinicians. The objective of the engagement process was to improve the quality of care through future organizational changes oriented towards the achievement of the adapted model of care emerged from this process.

## Methods

We defined a strategy to include the participation of patients and caregivers in the elaboration of the Metropolitana Nord Community-based Integrated Care Programme to People with Complex Chronic Conditions (Programa ProPCC MetroNord Institut Català de la Salut) [23], tailored to people with complex chronic conditions and high social and care needs, that would identify key clinical actions for healthcare and social care staff to perform at different points throughout the entire trajectory of care. The initiative was developed in the framework of the quality improvement process led by the Metropolitana Nord Chronic Care Management Team, from the Gerència Territorial Metropolitana Nord, of the Institut Català de la Salut. Its main aim was planning and evaluating innovative integrated care strategies for patients with frailty and multimorbidity attended by our teams located in the north of the metropolitan area of Barcelona, Catalonia. The project involved more than one hundred people from the town of Badalona (third-largest city in Catalonia) January through November 2018.

This strategy comprised two components: (C1) a qualitative study to explore patients’ and caregivers’ experiences and views; and (C2) a task group with professionals to validate evidence-based key actions and to adapt them to their local contexts.

### C1. Capturing Patients’ and Caregivers’ Experience

Following the precepts of a contributory project [24] to integrate the patients’ and caregivers’ views in designing the programme, we performed a qualitative study with the aim to analyse patients’ and caregivers’ experience regarding the services provided by the local system to meet their high needs due to complex chronic conditions.

#### Sampling and data collection procedures

We recruited a convenience sample of people with complex chronic conditions (including patients in need of end-of-life care), caregivers supporting or having supported them (in some cases the patient had recently died), and representatives of patient associations (oncologic and progressive non-oncologic diseases).

We established an interview schedule for sequentially combining data collection and initial analysis of both group and individual interviews. An interview guide was designed to ask participants about their experiences throughout the entire trajectory of care, with specific questions about (1) high needs impact, (2) care plans, (3) management of crises, (4) transitional care, and (5) end-of-life care. The interviews were adapted to the different types of participants (patients, caregivers, and professionals).

Interviewers recorded their observations on a data collection form specifically designed for this purpose. All interviews and group discussions with patients and caregivers were recorded and later transcribed.

Two researchers (MM and MU) contacted frontline staff from 11 Primary Care Centres to identify candidates who had previously been identified and registered in the electronic health record with the labels CCP or ACD, and with interest in being involved with the elaboration of the new programme. Physicians, nurses, and social workers from these centres, and linked to our group (they were participants of the task group with professionals), contacted patients and caregivers from their surgeries (by a phone call or in the context of a visit for any clinical or social reason). All candidates were informed that they were invited to participate in this project as part of a quality improvement plan. Therefore, candidates agreeing to participate signed an informed consent document authorizing the investigators to register and use the information derived from their participation. ***Table 1*** outlines the characteristics of the patients and caregivers recruited from the different primary care centres.

**Table 1:** Characteristics of patients and caregivers from different primary care centres. Onc: Oncological diagnostic; Non-onc: Non-oncological diagnostic; CCP: Complex chronic patient, ACD: Advanced chronic disease; we considered old man/woman if aged ≥65.


	ONC CCP	NON-ONC CCP	CARER OR FAMILY MEMBER ONC CCP	CARER OR FAMILY MEMBER NON-ONC CCP	ONC ACD PATIENT	NON-ONC ACD PATIENT	CARER OR FAMILY MEMBER ONC ACD	CARER OR FAMILY MEMBER NON-ONC ACD	FAMILY MEMBER AFTER ACD PATIENT DEATH

*Centre 1*	Old man						Young woman		

*Centre 2*			Old woman		Young man				Young woman

*Centre 3*	Young man							Old woman	

*Centre 4*			Young woman			Old man			

*Centre 5*		Young woman					Old man		Old woman

*Centre 6*				Young man	Old woman				

*Centre 7*		Old woman						Young man	

*Centre 8*				Old man		Young woman			Old man

*Centre 9*			Old Man				Young woman		

*Centre 10*				Young woman				Old man	

*Centre 11*	Old woman					Young man			Young women


To gather information about patients’ and caregivers’ experiences, as well as facilitating triangulating their views, we combined three techniques:

- focus groups (one for complex chronic patients and another for their caregivers).
- semi-structured interviews with 19 patients and caregivers (including patients with advanced illness, their caregivers -often interviewed alongside the patients-, and caregivers of patients who had died recently).
- in-depth interviews with representatives of 3 associations supporting patients with progressive disease (amyotrophic lateral sclerosis, dementia, and cancer).

### C2. Input from Healthcare and Social Care Professionals

To incorporate professionals’ perspective and knowledge in the programme design, we created a focus group for each of the five stages of the care trajectory (identification of high needs, care planning, crises management, transitional care, and end-of-life care). Each group comprised 9 to 11 healthcare and social care individuals from our institution with different backgrounds and professional roles providing care in different contexts (primary and community care, intermediate care and hospital care). ***Table 2*** reports the composition of the different groups.

**Table 2:** Composition of focus groups with health and social care staff.


GROUP	PROFESSION/DISCIPLINE/SPECIALITY	UNIT/SETTING

**Group 1 Identification of high needs**	Nurse Nurse case manager General Practitioner General Practitioner Social worker Physician Geriatrician Pneumologist Internist	Outpatient primary care Home-based primary care Outpatient primary care Home-based primary care Outpatient primary care Emergency department Outpatient hospital care Outpatient hospital care Hospital-at-home

**Group 2 Care planning**	Nurse General Practitioner General Practitioner Social worker Cardiologist Occupational therapist Internist Administrative staff General Practitioner Director	Outpatient primary care Outpatient primary care Outpatient primary care Outpatient primary care Outpatient hospital care Hospital ward Hospital ward Outpatient primary care Home-based primary care Primary care team

**Group 3 Crises management**	Nurse case manager General Practitioner General Practitioner General Practitioner Pneumologist Physician Internist Nurse Internist Social worker Nurse	Home-based primary care Home-based primary care Acute home care Acute home care Day hospital Emergency department Hospital ward Day hospital Hospital-at-home Outpatient primary care Outpatient primary care

**Group 4 Transitional care**	Nurse case manager General Practitioner Social worker Nurse Administrative staff Coordinator Nurse Nurse Social worker	Home-based primary care Home-based primary care Hospital ward Hospital liaison Home-based primary care Hospital-at-home Outpatient hospital care Outpatient primary care Outpatient primary care

**Group 5 End-of-life care**	Nurse case manager General Practitioner Social worker Palliative care nurse Geriatrician Geriatrics nurse Palliative care physician Physician Director Physician	Home-based primary care Outpatient primary care Outpatient primary care Home-based palliative care Hospital-at-home Hospital ward Outpatient hospital Acute home care Primary care team Home-based primary care


**Figure 1:**
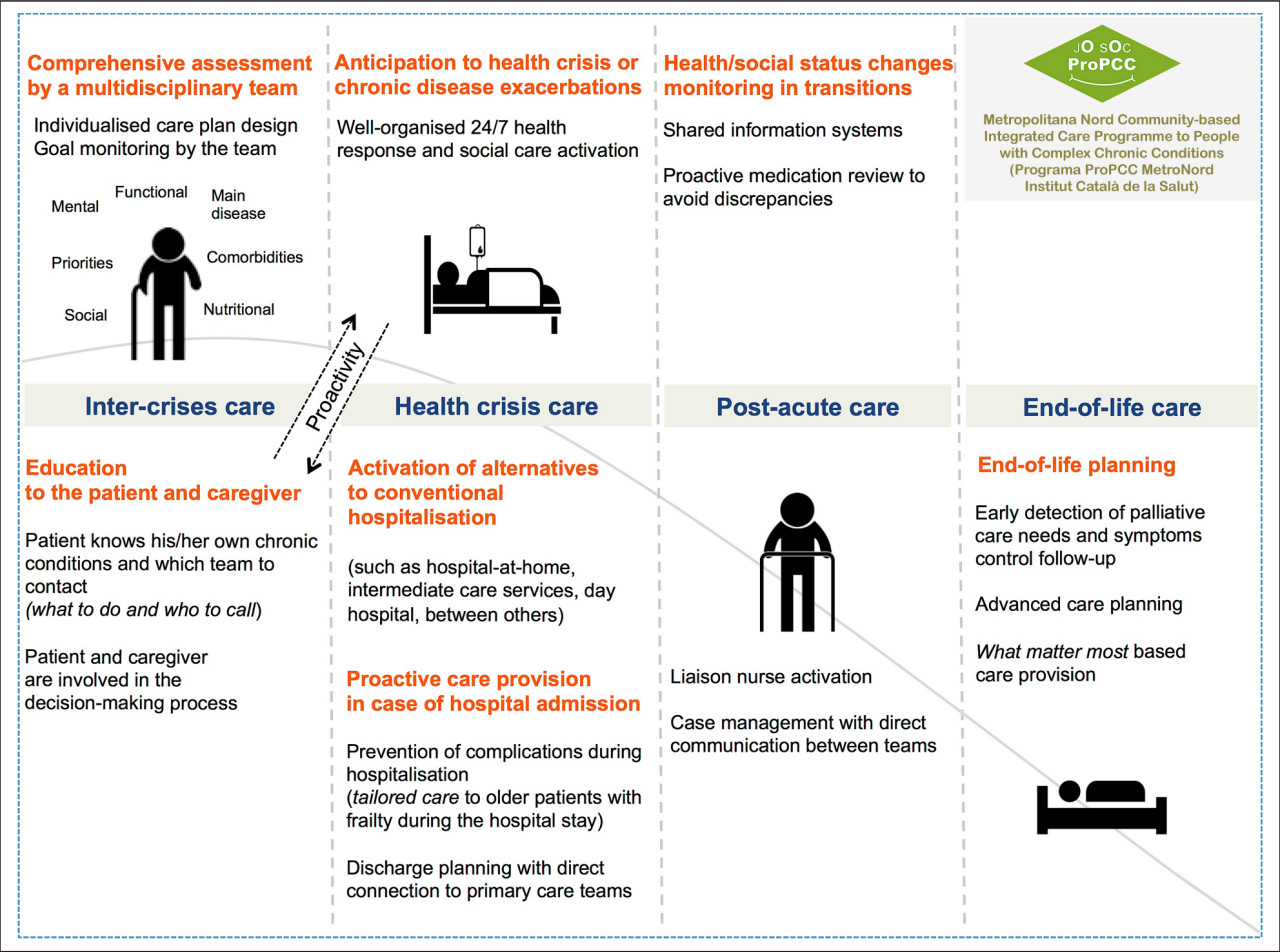
Evidence-based clinical practices for people with complex chronic conditions, in which health and social professionals agreed.

Each group met in two to four sessions. In each session, two moderators from the research team started an open discussion on staff members’ experiences providing care in their care trajectory stage. With the aim of defining evidence-based actions for their stages of the care trajectory, participants listed clinical actions that could fulfil the needs of patients and caregivers. The clinical leaders of this project (MM and RM) validated the main evidence for these actions for each stage [25,26,27,28,29,30]. They used high-quality research selected based on a US Institute of Medicine’s approach on successful models of comprehensive care for older adults with chronic conditions [31,32]. Fifteen urging models were considered, including: interdisciplinary primary care [33], models that supplement primary care, transitional care, models of acute care in patients’ homes, nurse-physician teams for residents of nursing homes, and models of comprehensive care in hospitals. According to these reviews, a graphic panel with evidence-based good clinical practices throughout the care trajectory, in which health care and social professionals agreed was developed (***Figure 1***).

### Analysis

#### Patient and caregivers’ experience

The transcripts of the recordings from the interviews and discussions were annotated with the observations recorded by each interviewer/moderator in the data collection forms. Next, we conducted a thematic analysis [34] of the transcripts, combining both an inductive and a deductive approach. First, initial codes (e.g. legal issues, emotional wellbeing, social barriers, beliefs, etc.) were derived from the content of the data themselves by one of the co-authors (CH). Then, these codes were grouped into themes reflecting the key aspects regarding the model’s definition (what was being done right, unmet needs, and each person’s priorities) after being discussed by the co-authors (CH, MM & MJU). Then, we combined the information gathered from all the sources about each aspect for each stage of the care trajectory, underlining key aspects and ideas, and placing special emphasis on priorities to facilitate their incorporation into the programme (***Table 3*** and **Annex 1**).

**Table 3:** Main characteristics of our programme.


DIMENSION	PROGRAMA PROPCC METRONORD INSTITUT CATALÀ DE LA SALUT

**Quality of information and communication**	Ensuring patients and caregivers understand the information provided

**Coordination and participation**	Ensuring coordination between caregivers and professionals in managing health and social needs Ensuring social services when needed Providing support to caregivers in managing situations

**Continuous healthcare and social support/accompaniment**	Ensuring patients and caregivers feel supported throughout the process Enabling patients to be attended at home (if adequate care is available) Adopting a patient-centred approach


Throughout the analytical process, we took care to ensure that discrepant views and priorities were reflected in the analysis (e.g., some patients prefer to be attended by a specialist, even if this means waiting, whereas others prefer to be attended immediately, even if this means being attended by a different professional). Following Braun & Clarke’s reflexive approach to thematic analysis [35], we contend the adequacy of the notion of saturation in this study, since we agree that meaning is generated and not excavated from data, therefore being inescapably situated and subjective. For the purpose of this case study, we decided to conduct all interviews and focus group planned in order to mitigate missing key themes, even though no new ones were identified especially towards the final interviews.

At the end of the process, the results were member-checked since all participants (patients, caregivers, and representatives of patients) received a letter from the researchers with adapted information related to the main conclusions of the process, highlighting their input in the programme at the end of the process.

#### Integrating healthcare and social care professionals’ experience

To decide which clinical actions should be included in the programme, two experts (RM & MM) reviewed the contributions of different professionals (physicians, nurses, social workers, administrative staff, occupational therapists, etc.) based on their experience and their knowledge in the care of vulnerable populations in the local area, comparing them to evidence-based actions.

The programme was designed by combining the information derived from the experiences of patients, caregivers, and professionals, taking care to ensure that the contributions of the different groups of participants could be traced and identifying where they were incorporated.

## Results

**Figure 2:**
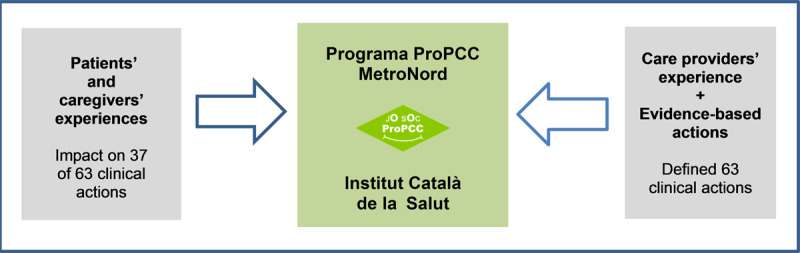
Designing person-centred integrated care considering patients’, caregivers’, and professionals’ experiences.

The ultimate result is an integrated care programme that includes 63 evidence-based actions, of which 37 (59%) were merged with actions derived from patients’ and caregivers’ experiences (***Figure 2***).

Below, we list the guiding principles of the programme, illustrating them with an excerpt from an interview or focus group with caregivers and participants in order to highlight their critical role in defining it.

**A. To ensure that patients and caregivers understand the information provided**

As this participant states regarding the caregiver, this priority corresponds to the demands of clear information and smooth communication between all those involved in the programme:

*“With just one explanation, the caregiver must become an expert on the topic” (GF_PCC)*.

**B. To coordinate the management of health and social needs between patients, caregivers, and professionals**

Linked to the necessity of fluid communication, this priority adds a more practical dimension of coordinating views, actions and decision making (e.g. remaining at home and avoiding nursing home admission), namely allow participation in care planning:

*“I fear he gets really sick and suffers. If this happens, I’d phone the nurse first thing. I trust him and he shares my views: avoid nursing homes” (EP3 MACA)*.

**C. To ensure that social services are available when they are needed**

This priority highlights the relevance of covering physical and environmental needs, as this caregiver points out regarding the importance of a wheelchair, even over more health-related issues:

*“I’ve never really needed anything. (…) Just once, I asked the government, the council, social care, etc. for an electric wheelchair and it was rejected. I am not talking about information but about something primordial. (…) that a 49-year-old can get out (…). I am not asking for luxury but for people’s needs being taken into account. The doctor is amazing but sometimes these other things are more important” (GF CAREPCC)*.

**D. To provide support to caregivers in managing situations**

This priority is related to the need to not only involve but also provide specific support for family members and caregivers, who often report feeling overwhelmed:

*“We need some help for the caregiver or a (different) caregiver some days a week… we are exhausted at all levels” (EP3 PCC)*.

**E. To ensure that patients and caregivers feel supported throughout the entire trajectory of care**

This priority relates to the emotional support, empathy, and respect needed throughout all stages of the care process, highlighting its dynamism (e.g. intense support to Primary Care teams by hospice at home teams may be needed in some cases of complexity) and the requirement of continuity of care and soft transitions:

*“The palliative care nurse came home only until I learned how to manage morphine regulation” (EP2 MACA)*.

**F. To enable patients to be attended at home (if care is available)**

This priority relates to a general (but not unanimous) claim of receiving effective, care by familiar, trusted staff at home. As this participant points out, home care is seen as a priority but acknowledging this may have some limitations and should not prevail over the patient’s wellbeing (e.g. feeling calmed):

*“I know my father will get worse, but we all want him to stay at home until the end if he’s calmed. He’s already been attended by the home care palliative care team once and it was great” (EP1 CAREPCC)*.

**G. To facilitate a patient-centred approach**

Somehow integrating the priorities listed above, this priority highlights the importance of respecting patients’ and caregivers’ values and preferences and allowing them to participate in care planning, even when these contradict established clinical advice (as in the excerpt below):

*“The nephrologist told me to go to the hospital for everything. I was told this (an inhaler) could harm my heart but to avoid going to the hospital, I chose this option. I told the nurse: “I found this, and I have done that” and she said: don’t do this more than three days but if you really need it, go ahead, it won’t hurt you and it can clean your lungs”, I did not want to get to the hospital at all” (GF PCC).*

## Discussion

Our research has identified actions and priorities that are similar to those outlined in reference projects from the United Kingdom: National Voices [14] and Oxford Picker Institute [16]. The main dimensions identified from the contributions of patients and caregivers in our study are those related to the quality of information and communication, coordination and participation, and continuous health and social support/accompaniment.

In recent years, various groups have involved patients in service planning. In a scoping review of 22 studies reporting the experience of patients with multimorbidity, van der Aa et al. [18] identified 12 categories of experience with the healthcare process, dividing problems into those considered system-related (burden of care, lack of organization, poor communication and insufficient access to care) and those considered professional-related (competencies, managing information, attitude towards vulnerable patients and carers, guidance, communication skills, holistic view, familiarity, and patient involvement). Shiotz et al. [19] conducted semi-structured qualitative interviews with 14 patients with multimorbidity, identifying three dimensions related with continuity (information continuity, management continuity, and relational continuity). These authors identified a dichotomy in patients’ reports about their care experience: on the one hand, patients reported many problems and areas in which care could be improved; on the other hand, however, they also reported that they were mostly satisfied with and thankful for the care they received. These authors conclude that patients like to be treated as a whole person by healthcare professionals with time who collaborate with other professionals across the system to provide specialized care to manage their complexity. They also underline the importance of medication management. In a multicentre quality study involving 172 patients with multimorbidity and caregivers, Kuluski et al. [17] sought to ascertain the priorities of patients and their caregivers regarding care. They found that the priorities were (a) feeling heard, appreciated, and comfortable; (b) having someone to count on; (c) having easy access to health and social care; (d) knowing how to manage health and what to expect; (e) feeling safe; and (f) being independent. To understand healthcare professionals’ perspectives about managing patients with multimorbidity, Sinnott et al. [30] synthesized the findings from 10 studies including a total of 275 general practitioners in 7 countries. They found that problematic aspects of managing these patients fell into four areas: the disorganization and fragmentation of healthcare, the lack of guidelines adapted to patients needing complex care, the difficulties involved in delivering patient-centred care, and barriers to shared decision-making.

Our work integrated the viewpoints of patients, caregivers, and professionals to design a new model of integrated care in a territory. We provided the means and opportunity for those living with complex chronic conditions, and their family members and caregivers, to express their views about how healthcare services can best meet their needs. Likewise, we considered it important to incorporate the viewpoints of the different professionals involved in caring for these patients into a new, person-centred approach to managing these high-need patients. The model resulting from this quality process (***Table 4***) is in the same line as advocated by Poitras et al. [25] based on their scoping review, which underlined the importance of promoting evidence-based decision making, adopting patient-centred approaches, enabling patient self-management, facilitating case/care management, promoting interdisciplinary approaches, developing training for healthcare professionals, and integrating information technology.

**Table 4:** Summary of the key actions of the programme.


**1. Identification of high needs**

Weekly multidisciplinary meetings in primary care centres and hospital to detect high need-patients

**2. Definition and provision of an individualized care plan**

Multidimensional assessment using Comprehensive Geriatric Assessment tools

Weekly multidisciplinary meetings in primary care centres:

Defining shared goals with patients

Defining therapeutic intensity level

Protocoled proactive visits

Health education on illness and care

Social needs assessment and service activation

Individualized care plan registers in electronic health record based on person values and priorities

**3. Management of health crises**

Centralized response to acute crises

Acute response goal <12 hours

Direct access to alternative to hospitalization resources

Case management with direct communication between units

**4. Transitional care**

Multidimensional assessment using Comprehensive Geriatric Assessment tools during hospitalization

Case management with direct communication between units during hospitalization

Care planning during hospitalization focused on return to home

Healthcare and treatment education

**5. End-of-life care**

Exploring what matters most and social resources for end-of-life care at home

Early detection of palliative care needs

Advanced care planning with patients and caregivers

Meetings every 2 weeks for collaboration between units in and-of-life care at home/nursing home


Several limitations and considerations related to the nature of the co-design process in people with complex chronic conditions and their caregivers were identified. Firstly, we found difficulties in the engagement process. Circumstances made it necessary to partially modify the strategy that had been planned. Although the focus groups provided ample useful information, attendance was lower than expected (only 6 patients and 5 family caregivers) because some patients’ conditions had worsened, so neither they nor their caretakers could attend. To ensure that all patients and caregivers could participate, we interviewed them in their homes, thus increasing the number of visits and prolonging data collection. Despite these drawbacks, this approach ensured a more representative selection of patients and caregivers by including those who were especially frail and complex. Secondly, we assume that the voluntary participation of people (patients and caregivers) with special motivation to collaborate in the care improvement process implies a bias related with the nature of our pragmatic approach. Thirdly, despite reporting to the participants the highlights of their input in the final version of the programme at the end of the process, more feedback is needed to monitor the adaptation of the programme to their views and needs. The fact of not including patients and caregivers in the design of key actions leads to an incomplete co-design process. This inclusion was considered too complex in our approach to the design of our study, due to cultural reasons and other organisational factors. We will take this situation in consideration for future updates of our care model strategy, based on the successful results of this initial experience.

Finally, we would like to highlight the impact of this quality process in the development of the new model. Through 2019 and the first trimester of 2020, the task continued with several meetings with directors and clinical leaders of the region to translate it into redesign service production, at a community level, and at intermediate care and acute hospital level. Key actions of the programme were used as a standard quality framework for the detection of high-need high patients and for the case management provision across the care continuum. Most units could modify their care without major structural changes in their teams, but Metropolitana Nord Primary Care teams and the Department of Geriatrics of Hospital Universitari Germans Trias i Pujol, decided to build new multidisciplinary case management units, with expert social and health staff, to improve to provision of person-centred care based on the care recommendations of the new programme. The validation of the model was interrupted due to the Covid-19 pandemic in 2020. We are working on completing the evaluation of the project, by following the ProPCC-Badalona cohort evolution [36].

### Lessons Learned and Future Vision

- Querying people with complex chronic conditions and their caregivers identified the following dimensions that should be prioritized in the elaboration of a more patient-centred approach to their care: the quality of information and communication, coordination and participation, and continuous support/accompaniment by social care and healthcare professionals. These priorities were converted into clinical actions that affected more than 50% of the actions in the evidence-based integrated clinical care programme elaborated in collaboration with healthcare and social care professionals from our institution.
- Involving the public in improving the process and organization of care requires the dedication of additional resources, mainly professionals’ time and knowledge, but the contributions of patients and caregivers help ensure that we take their experiences into account in designing and improving care programmes, to meet their needs in accordance with their preferences and priorities.
- Sharing this rigorous practical collaborative project has helped clinical and organizational leaders and professionals to rediscover the value of prioritizing patients’ priorities. We are aware that this has not been a pure co-production process, but a first step for our institution towards service co-designing of complex interventions’ care, tailored to high-need vulnerable individuals and populations.
- Our next step will be engaging patients and caregivers in the systematic evaluation of the programme, combining qualitative and quantitative methods to measure and monitor patients’ needs and wellbeing.

## Additional File

The additional file for this article can be found as follows

10.5334/ijic.5653.s1 Annex 1. List of priorities to convert in actions for the entire care trajectory.
