# Supplementary material for: Designing a Person-Centred Integrated Care Programme for People with Complex Chronic Conditions: A Case Study from Catalonia
Source: Int J Integr Care. 2021 Nov 25;21(4):22. doi: 10.5334/ijic.5653 (PMC8622001; doi:10.5334/ijic.5653)
Supplement: Annex 1. — List of priorities to convert in actions for the entire care trajectory. [file ijic-21-4-5653-s1.pdf]

Annex 1 List of priorities to convert in actions for the entire care trajectory

Transversal (15 actions)

Patients and caregivers need psychological support
Patients and caregivers need to be heard
Patients and caregivers need information delivered in plain language as well as guidance and support
Professionals must be well trained
Professionals must be knowledgeable
Professionals must be able to empathize with patients
Caregivers need support
Patients and caregivers need more information about available resources; families need guidance
Patients need support for losses occurring throughout the course of disease
Patients need to be empowered
Family members need to be able to communicate with professionals without the presence of the patient
Communication between hospital professionals and families must be improved
Good interpersonal treatment and manners are important for perceived quality
Caregivers need more support
Caregivers need good information

Detection of high needs (2 actions)

The patient should feel accompanied during diagnosis and explanations
Caregivers should receive the right amount of information (neither too much, nor too little)

Follow-up care plan (18 actions)

Caregivers need rapid, home-based solutions about attending family members (advice, tips, strategies)
Patients prefer the assigned professionals, even if they have to wait
Nursing and medical care and follow-up at home are deeply appreciated
Hospital and primary care professionals need to coordinate closely
Patients need help at home (financial support for caregivers, volunteers)
Steps should be taken to avoid sending patients to nursing homes
Family members need breaks from caring for patients
Social work must be systematized
Caregivers need courses about how to provide care
Caregivers groups should be created for patients; there should be open sessions where family members can express their needs
There needs to be a programme of activities for families
Care must be coordinated with the hospital: outpatient visits must not be cancelled without advance notification
Information, advice, recommendations, and guidelines should be provided in writing
Early identification and communication of symptoms strategies should be available for patients and caregivers
Patients' home must be adapted to their special needs and limited mobility
Patients should have the appropriate technology, including GPS tracking
Caregivers should have a way out
Caregivers should have sufficient resources to attend patients as their disease progresses

Crises management (4 actions)

The system should avoid unnecessary trips to the hospital
Patients prefer hospital care during crises because treatments are done there
Patients prefer to be attended at home during crises, provided the treatment is the same in both places

Original manuscript

Patients need to have a 24-hour hotline where they can consult professionals and solicit help at home if necessary

Transitional care (18 actions)

Hospital, primary care, and home-based care must be coordinated
Patients must have the technical support they need to return home after hospitalization

End-of-life care (6 actions)

Support groups should be created to facilitate mourning
Family members/caregivers have no preferences for the place where end-of-life takes place: wherever the patient is most comfortable and his or her needs are met best
It is essential to avoid suffering at the end of life
Patients prefer to be admitted to nursing homes than to become a burden for the family
Patients prefer to die at home
Professionals should take actions to get to know the patient and family before the end of life

Original manuscript
